# Pre-or co-SARS-CoV-2 Infections Significantly Increase Severe Dengue Virus Disease Criteria: Implications for Clinicians

**DOI:** 10.3390/pathogens13070573

**Published:** 2024-07-10

**Authors:** Moeen Hamid Bukhari, Esther Annan, Ubydul Haque, Pedro Arango, Andrew K. I. Falconar, Claudia M. Romero-Vivas

**Affiliations:** 1 Department of Statistics, Quaid-i-Azam University, Islamabad 45320, Pakistan; 2 Centre for Health and Wellbeing, School of Public and International Affairs, Princeton University, Princeton, NJ 08544, USA; 3 Rutgers Global Health Institute, New Brunswick, NJ 08901, USA; 4 Department of Biostatistics and Epidemiology, School of Public Health, Rutgers University, Piscataway, NJ 08901, USA; 5 District Department of Health of Barranquilla, Barranquilla 080003, Colombia; 6 Laboratory of Tropical Diseases, Department of Medicine, Health Division, Universidad del Norte, Barranquilla 080003, Colombiaclromero@uninorte.edu.co (C.M.R.-V.)

**Keywords:** dengue fever, severe dengue, dengue coinfection, Colombia, COVID-19, SARS-CoV-2

## Abstract

Few studies have investigated whether SARS-CoV-2 infections increase the incidence of dengue haemorrhagic fever/shock syndrome (DHF/DSS) and/or severe dengue (SD) in dengue virus (DENV)-infected patients. This study was performed on a site with high incidences of classical dengue, but relatively few DHF/DSS or SD cases as defined by the WHO 1997 or 2009 criteria, respectively. Clinical, haematological/biochemical, and viral diagnostic data were collected from febrile patients before, during, and after the COVID-19 epidemic to assess whether (a) DENV-infected patients with prior SARS-CoV-2 infections or (b) DENV-SARS-CoV-2-co-infected patients had increased incidences of SD/DHF/DSS using logistic regression and machine learning models. Higher numbers of DHF/DSS/SD occurred during the COVID-19 epidemic, particularly in males and 18–40-year-olds. Significantly increased symptoms in the DENV-SARS-CoV-2-co-infected cases were (a) haemoconcentration (*p* < 0.0009) and hypotension (*p* < 0.0005) (DHF/DSS and SD criteria), (b) thrombocytopenia and mucosal bleeding (DHF/DSS-criteria), (c) abdominal pain, persistent vomiting, mucosal bleeding, and thrombocytopenia (SD warning signs) and (d) dyspnoea, but without fluid accumulation. DENV-infected patients with prior SARS-CoV-2 infections had significantly increased incidences of thrombocytopenia (DHF/DSS-criteria) and/or abdominal pain and persistent vomiting and also thrombocytopenia (SD warning signs), but without significant haemoconcentration or hypotension. DENV-SARS-CoV-2 co-infections significantly increased the incidence of DHF/DSS/SD, while DENV-infected patients with prior SARS-CoV-2 infections displayed significantly increased incidences of thrombocytopenia (DHF/DSS-criteria) and three important SD warning signs, which are therefore very important for health workers/clinicians in assessing patients’ DHF/DSS/SD risk factors and planning their optimal therapies.

## 1. Introduction

Dengue virus (DENV), with four discrete serotypes, has become endemic in most tropical and subtropical countries of the world and globally causes an estimated 100–400 million cases of infections annually [1]. Dengue fever (DF) usually occurs as a self-limiting febrile disease, termed classical DF; however, it can also be associated with severe life-threatening manifestations like dengue haemorrhagic fever and shock syndrome (DHF/DSS). The clinical diagnostic criteria for DHF and DSS have been fully defined by the World Health Organization [2] and have been used as the reference criteria for distinguishing between DF, DHF, and DSS cases. As such, the hallmark for DF and DHF discrimination has been: (a) vascular leakage leading to haemoconcentration through >20% increased haematocrit values above the average for age, sex, and population and fluid accumulation as observed by pleural effusion, ascites, or hypo-proteinuria, (b) increased bleeding through a range of symptoms including a positive tourniquet test, petechiae, purpura, mucosal or injection-site bleeding, or haematemesis/melena, (c) severe thrombocytopenia (<100,000/μL), and, often, (d) hepatomegaly (>2 cm) [2]. DHF cases were further subdivided into four grades (DHF I to IV), where DHF III and IV were further classified by narrowed (<20 mm Hg) pulse pressure or hypotension (DHF III) or profound shock (DHF IV). 

Subsequently, the WHO altered the definition of DHF/DSS to ‘severe dengue’ (SD), which also included SD warning signs [3]. The latter was introduced to include the range of different organ diseases associated with DENV infections and other results resulting from vascular leakage fluid accumulations, such as acute respiratory distress syndrome (ARDS). Although generally accepted, these new SD classifications based on severe vascular leakage, bleeding, and organ damage (including encephalopathy) and SD warning signs have also been criticised due to not including established discriminatory values for thrombocytopenia, elevation of haemoconcentration (haematocrit), or pulse pressures as was provided in the WHO 1997 criteria [4,5], while an early meta-analysis claimed that the WHO 2009 criteria were more reliable for identifying severe cases [6]. In other studies, however, (a) both the WHO 1997 and 2009 criteria were equally effective, but the WHO 2009 criteria were also better for the identification of the most severe cases [7]; (b) the WHO 1997 criteria for plasma leakage and hemodynamic compromise was suggested to be re-introduced into the WHO 2009 SD criteria [5]; and (c) the WHO 2019 criteria had a low sensitivity for patients requiring urgent care and predicting mortality, and the authors strongly suggested that thrombocytopenia-related bleeding should be re-introduced into the WHO 2009 SD criteria, together with the effects of particular chronic diseases [8]. As such, it is therefore prudent to employ both the WHO 1997 and 2009 DHF/DSS/SD criteria for application to DENV-infected patients to subsequently identify and employ the appropriate therapies for all life-threatening DENV cases. 

The COVID-19 pandemic, caused by SARS-CoV-2, led to more than 768 million infections and 7 million deaths throughout the world, and caused a large number of deaths due to acute and chronic disease (long COVID-19) in patients [9]. Further, acute disease was associated with acute respiratory distress and hypoxia; many of the symptoms of SARS-CoV-2 infections are similar to those displayed by classical DF patients [9,10], thereby making clinical differential diagnosis difficult, but while acute dyspnoea (ARDS: acute respiratory distress syndrome) often occurs in symptomatic acute SARS-CoV-2 cases, it may also lead to chronic pulmonary disease as well as multi-organ disease symptoms in ‘long-COVID’ cases [11]. Despite the expected high incidence of DENV-SARS-CoV-2 co-infections in DENV endemic areas, only a few studies have been performed on their outcomes [12,13]. To the best of our knowledge, there have been no studies performed to also assess whether the DENV-infected patients who were previously infected with SARS-CoV-2 infections displayed significantly increased incidences of WHO 1997 DHF/DSS or WHO 2009 SD and SD warning sign criteria despite their importance for health workers. 

Barranquilla is a principal seaport on the Caribbean coast of Colombia where all four DENV serotypes are endemic and cause large numbers of self-limiting DF throughout the year, but where DHF/DSS/SD cases are rare [14,15]. As such, this study site was an ideal location to investigate whether the COVID-19 epidemic increased the incidence of DENV disease when assessed using both the WHO 1997 DHF/DSS-defined criteria and the WHO 2009 SD and SD warning sign criteria in DENV-SARS-CoV-2-co-infected patients or, very importantly and uniquely, also in DENV-infected patients who had previously encountered SARS-CoV-2 infections.

## 2. Methods

### 2.1. Setting and Population

Febrile patients were recruited from hospitals in the Caribbean coastal city of Barranquilla, Colombia, between 2018 and 2022 and were only included in this study if they were then confirmed as anti-dengue virus (DENV) IgM ELISA-positive for dengue virus and also had a high (>38 °C) fever and at least one of these clinical symptoms: (i) retro-orbital pain, (ii) myalgia, (iii) headache, or (iv) rash. Patients with suspected SARS-CoV-2 infections (COVID-19 disease) were confirmed using a specific reverse-transcription polymerase chain reaction (RT-PCR). 

The DENV-infected cases were then assessed for symptoms of serious disease using the combined WHO 1997 dengue haemorrhagic fever/dengue shock syndrome (DHF/DSS) criteria [2] and the updated WHO 2009 severe dengue (SD) with SD warning signs [3], which included: (i) gastrointestinal effects (e.g., abdominal pain and persistent vomiting) (WHO 2009 SD warning sign), (ii) plasma leakage, as observed by haemoconcentration (increased haematocrit values or other parameters (e.g., pleural effusion/ascites fluid accumulation, which may have possibly led to dyspnoea/acute respiratory distress syndrome (ARDS) (WHO 1997 DHF/DSS and WHO 2009 SD criteria)), (iii) thrombocytopenia (WHO 1997 DHF/DSS or WHO 2009 SD warning sign criteria), (iv) shock/hypotension (WHO 1997 DSS and WHO 2009 SD criteria), (v) moderate bleeding (positive tourniquet test or spontaneous bleeding from a range of sites such as the mucosa) (WHO 1997 DHF/DSS criteria), (vi) severe bleeding (WHO 1997 DHF/DSS and WHO 2009 SD criteria), or (vii) severe organ disease, particularly liver disease supported by hepatomegaly and increased liver-specific (ALT and AST) enzymes (WHO 1997 DHF/DSS and WHO 2009 SD criteria). 

Patient (demographic and clinical) information was registered by each of the hospitals in a case report form for dengue and SARS-CoV-2 infections as part of the national surveillance system for public health (SIVIGILA). For this study, we reviewed the medical records of all DENV-infection-confirmed cases that occurred before, during and after the COVID-19 epidemic in Barranquilla to investigate whether SARS-CoV-2 infections resulted in significantly increased incidences of DHF/DSS/SD cases, based on the WHO DHF/DSS 1997 and WHO SD and SD warning sign criteria due to DENV-SARS-CoV-2 co-infections or in DENV-infected cases who had previously encountered SARS-CoV-2 infections.

### 2.2. Statistical Analysis

In the descriptive analysis, frequencies, and percentages (%) were used to present the qualitative variables. The chi-square test and Fisher exact test were performed to compare the percentages between the co-infected and non-co-infected cases, between SARS-CoV-2 positive and negative cases, and between severe (DHF/DSS/SD) and non-severe dengue cases, as appropriate.

Logistic regression analysis was first performed to identify the more important clinical manifestations and laboratory findings associated with co-infected patients, and second the clinical symptoms that influenced DENV infection severity in patients. In multivariate analysis, the co-infected model was further analysed by adding the clinical manifestations and laboratory finding variables simultaneously and eliminating the less significant variables (i.e., *p* > 0.05) from the model and the final model retained only significant variables. As age has a crucial role in infectious diseases, we integrated age into the final model. Further, multivariate analyses were conducted to compare symptomatic patterns between DENV-infected patients with previous SARS-CoV-2 infection history. The fitness of models was evaluated using the Bayesian Information Criterion (BIC) and the variance inflation factor (VIF) was used to evaluate multicollinearity in the models. Further, odds ratios (crude odds ratios (crude ORs) and adjusted odds ratios (aORs)) were estimated with 95% confidence intervals (95% CIs) to quantify the associations between independent factors with diseases in all models.

Machine learning techniques including decision tree (DT) and random forest (RF) models were utilised to find the key risk factors for DHF/DSS/SD (severe dengue) patients. By using random assignment, we conducted a split-sample validation and a facilitation model was constructed using a training sample (80%) that was tested on a hold-out sample (20%). We set the parent node to 30 and the child node to 10 for the minimum number of cases. Furthermore, we pruned the tree to avoid overfitting. The result’s validity indicated the unbiased assessment of fitted models using training datasets. Thus, the index including positive predictive value, negative predictive value, sensitivity, specificity, accuracy and AUC were used to validate the models. 

Based on the selected model, we computed the feature importance on permuted out-of-bag (OOB) samples for DHF/DSS/SD (severe dengue) patients by using the mean decrease accuracy method. The higher mean decreased accuracy indicated the more important features. All analyses were performed in the R language software (version 4.4.0) [16]. The R package CARET [17] was used for logistic regression models and RF and RPART [18] for DT.

## 3. Results

### 3.1. General Characteristics

Of 3611 DENV IgM-positive ELISA confirmed cases, (a) 49.99% (*n* = 1805) were male and 50.01% (*n* = 1806) were female, (b) 79.12% (*n* = 2857) belonged to the 18–40-year-old age group, and (c) 36.44% (*n* = 1316) of these patients belonged to the lowest socio-economic stratum, stratum 1 (Table 1). Overall, the number of hospitalized DENV-infected individuals increased from 373 before the COVID-19 virus pandemic (2018–2019) to 1687 during the COVID-19 pandemic period (2020–2022). The highest average of clinical characteristics before/during the COVID-19 pandemic were fever (100/100%), headache (89.00/85.69%), myalgias (84.83/81.87%) and arthralgia (77.17/71.37%). Interestingly, these IgM-captured ELISA-confirmed DENV-infected patients displayed increased incidences of symptoms when they occurred before the COVID-19 pandemic compared to those DENV-confirmed infections encountered during the COVID-19 pandemic. As such, these increased symptoms included retro-orbital pain (51.00/45.83%), rash (41.17/28.79%), abdominal pain (42.50/35.44%), vomiting (30.83/22.82%), diarrhoea (17.00/12.55%), hypotension (14.83/6.84%), bloody mucus (6.83/4.68%), haemoconcentration (increase haematocrit) (15.50/17.70) and liquid accumulation (9.83/4.88%) in these DENV-confirmed cases before the COVID-19 pandemic (Table 1). Severe dengue patients were more likely to have abdominal pain (50.2%; *p*-value < 0.01), vomiting (34.4%, *p*-value = 0.04), hypotension (13.3%; *p*-value = 0.04), hepatomegaly (12.6%; *p*-value = 0.04), dyspnoea (34.4%; *p*-value < 0.01) and bloody mucus (10.9%; *p*-value = 0.03) compared to non-severe dengue patients (Table S1). Among 3611 patients, 2208 (61.1%) had low platelet levels, 833 (23.1%) had low white blood cell (WBC) counts and 779 (21.0%) had raised ALT concentrations and 659 (19.1%) had raised AST concentrations (Table S2).

### 3.2. Statistical Comparison of the Symptoms of Patients with DENV-Confirmed Infections Who Were SARS-CoV-2 Negative with Those Encountering Ongoing DENV-SARS-CoV-2 Virus-Confirmed Co-Infections

Importantly, 517 IgM ELISA-positive acute DENV-infected patients were also confirmed to have SARS-CoV-2 infections using the specific RT-PCR and were therefore confirmed DENV-SARS-CoV-2-co-infected patients, while 1089 tested negative for SARS-CoV-2 (i.e., non-coinfections) (Table 2). When compared to DENV-infected patients who were COVID-19 RT-PCR negative, the DENV-SARS-CoV-2-co-infected cases showed statistically increased incidences of: (i) headache (87.04/86.41: *p* ≤ 0.0001), (ii) arthralgia (75.24/73.65%: *p* ≤ 0.0001, (iii) rash (31.33/29.48%: *p* = 0.005), (iv) hypotension (5.41%/3.39%: *p* = 0.0005), (v) haemoconcentration (4.26/3.95%: *p* = 0.0091), (vi) dyspnoea (34.4%/5.6%: *p ≤* 0.001) and reduced platelet numbers (38.88/38.29%: *p* ≤ 0.0001). In contrast, the DENV-confirmed cases showed statistically increased incidences of (i) myalgia (83.56/82.59%: *p* ≤ 0.0001), (ii) abdominal pain (34.62/33.66%: *p* ≤ 0.0001), (iii) vomiting (21.30/17.41%: *p* ≤ 0.0001), (iv) drowsiness (4.78/2.32%: *p* ≤ 0.0001) and bloody mucus (2.39/1.35%: 2.39/1.35%: *p* ≤ 0.0001) (Table 2).

### 3.3. Clinical Symptoms of IgM-Captured ELISA-Confirmed DENV-Infected Patients with or without Prior Confirmed SARS-CoV-2 Infections

A total of 566 DENV IgM-captured ELISA-confirmed patients reported prior SARS-CoV-2 RT-PCR-confirmed infections and whose symptoms were compared with DENV-confirmed patients with no prior SARS-CoV-2 infection (Table S3). The significantly higher incidences of DENV patients with previous SARS-CoV-2 infections displayed headache (84.45%/84.24%: *p =* 0.0005), hypotension (12.5%/5.91%: *p =* 0.0376), dyspnoea (32.3%/27.58%: *p ≤* 0.001), haemoconcentration (14.8%/10.34%: *p =* 0.0418) and liquid accumulation (5.65%/1.97%: *p =* 0.0520) (Table S3). From the logistic regression results, the significant factors associated with dengue patients with a previous history of SARS-CoV-2 infections were fever, headache, myalgia, arthralgia, abdominal pain, dyspnoea, rash, reduced platelets, haemoconcentration and hypotension (Table S7). All VIF values (<10) indicate that there is no multicollinearity problem in the model (Table S8).

### 3.4. Factors Associated with DENV-SARS-CoV-2 Co-Infections

In the bivariate logistic model, fever, headache, myalgia, abdominal pain, arthralgia, fatigue, dyspnoea, hypotension and haemoconcentration were statistically significantly associated with DENV-SARS-CoV-2-co-infected cases, and the laboratory findings including platelets, ALT, and AST were statistically significant with DENV-SARS-CoV-2 co-infections (Table 3). The multivariate analysis identified that the significantly associated symptoms of DENV-SARS-CoV-2 were fever (adjusted odds ratio (aOR) 1.47; 95% CI, 1.07–2.02), headache (aOR 1.58; 95% CI, 1.01–2.48), myalgia (aOR 2.09; 95% CI, 1.73–2.53), abdominal pain (aOR3.41; 95% CI, 2.73–4.28), arthralgia (aOR 1.5; 95%CI, 1.04–2.18), dyspnoea (aOR 1.31; 95% CI, 1.11–1.68), hypotension (aOR 1.4; 95%CI, 1.15–1.71), haemoconcentration (aOR 1.31; 95% CI, 1.08–1.6) and reduced platelets (aOR 1.45; 95% CI, 1.08–1.94) (Table 3). The VIF values of the multivariate logistic models were less than 10, indicating no multicollinearity issue in the model (Table S4). The multivariate age-adjusted model confirmed seven independent indicators of DENV-SARS-CoV-2 co-infection including fever (aOR 1.67; 95%CI 1.19–2.06), headache (aOR 1.72; 95%CI 1.0–2.99), fatigue (aOR 2.01; 95%CI 1.54–2.61), hypotension (aOR 1.52; 95%CI, 1.18–1.96), dyspnoea (aOR 1.47; 95%CI 1.14–1.89), haemoconcentration (aOR 1.42; 95%CI 1.1–1.82) and platelets (aOR 1.76; 95%CI 1.15–2.71) (Table 3). All of the VIF values for the age-adjusted model were less than 10, implying no multicollinearity issue (Table S5).

### 3.5. Important Features of DHF/DSS/SD Cases

**Figure 1:**
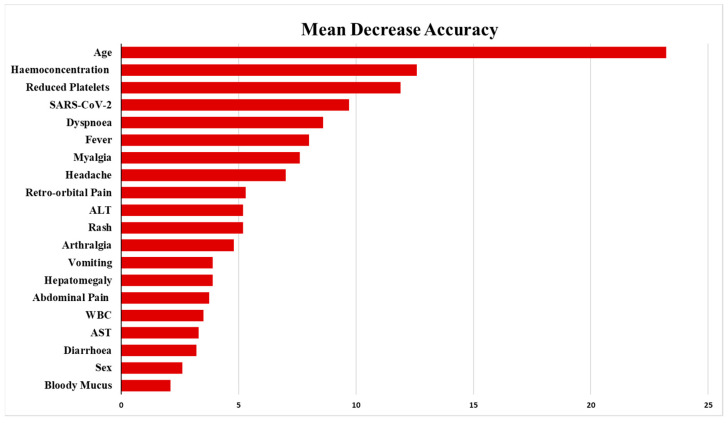
Identification of the important features of DHF/DSS/SD in patients using the random forest model. The OOB error rate is 14.5% with the number of trees (ntrees) (500) and 6 nodes tested (mtry).

The most important factors for DHF/DSS/SD were age, dyspnoea, reduced platelets, SARS-CoV-2 co-infection, and haemoconcentration (Figure 1). The statistical results of machine learning techniques indicated that the RF model performed better than the DT model in both the training and testing stages. The accuracy and AUC values for RF and DT are assessed as (0.85, 0.90) and (0.80, 0.86), respectively (Table S6). Moreover, after adjusting the multivariate logistic model for DHF/DSS/SD, the model identified the nine independent indicators for DHF/DSS/SD as fever, headache, retro-orbital pain, myalgia, arthralgia, abdominal pain, dyspnoea, haemoconcentration, previous SARS-CoV-2 infection and reduced platelet numbers (thrombocytopenia) (Table 4). 

## 4. Discussion

The main findings of this study were firstly that DENV-SARS-CoV-2 co-infections resulted in a significantly increased incidence of DHF/DSS/SD cases based on both the WHO 1997 DHF/DSS and the WHO 2009 SD with SD warning signs criteria in an area where severe DENV disease is relatively rare. Importantly, the most salient discriminatory criterion in both guidelines is vascular leakage, which was evident due to the significantly increased incidence of haemoconcentration (DHF/SD criteria), which also led to a significantly increased incidence of hypotension (DSS/SD criteria) in these DENV-SARS-CoV-2-co-infected patients. These results are, therefore, in agreement with those published previously [12,13]. Secondly, previous SARS-CoV-2 infections were also associated with a significantly increased incidence of thrombocytopenia and mucosal bleeding using the WHO 1997 DHF/DSS criteria, while they displayed significant incidences of important SD warning signs of abdominal pain, persistent vomiting, and reduced platelet numbers (thrombocytopenia). As such, we believe that this is the first report of the WHO 1997 or WHO 2009 DHF/DSS/SD or SD warning signs criteria being assessed and shown to be significantly increased in DENV-infected patients due to previous SARS-CoV-2 infections. The results strongly suggest that health workers need to question DENV-infected patients as to whether they have previously encountered confirmed or suspected SARS-CoV-2 infections due to these findings and to re-introduce thrombocytopenia and other known risks due to particular chronic diseases into the WHO SD criteria, as was suggested previously [8]. While a significantly increased incidence of dyspnoea/ARDS was identified in the DENV-SARS-CoV-2-co-infected patients, it is common in symptomatic SARS-CoV-2 patients but is also a WHO 2009 SD warning sign. The lack of significant fluid accumulation despite the significantly increased incidence of abdominal pain in the DENV-SARS-CoV-2-co-infected patients, however, suggested that the dyspnoea/ARDS incidence was mainly due to their SARS-CoV-2 infection. Dyspnoea/ARDS was not, however, significantly increased in the DENV-infected patients who had encountered previous SARS-CoV-2 infections. It would, therefore, be very interesting to establish whether patients with multi-organ symptoms, including pulmonary disease, due to chronic ‘long-COVID’ [11] have statistically increased incidence of DHF/DSS/SD disease or other SD warning signs and should, therefore, also be assessed in further studies.

There was a higher incidence of DENV infections as well as DENV-SARS-CoV-2-co-infected cases in patients from the lower (1–4) socio-economic strata than patients from strata 5 and 6, which was likely to be due to residents residing in houses rather than apartments, which increased the risk of reported DENV infections [19,20]. This is especially due to higher numbers of large-volume domestic water-storage containers [21], which are the principal breeding sites for its vector species, Aedes aegypti [14]. As such, the Barranquilla Health Authorities have consistently reported that neighbourhoods within these lower economic strata have much higher incidences of human DENV infections through their efficient national guidelines [22] and have, therefore, been the focus of surveillance and control efforts in this city [23,24] and elsewhere in Colombia [23,25]. Patients with DHF/DSS/SD symptoms from any of these six socio-economic strata are, however, extremely likely to report to clinics and hospitals and receive appropriate clinical and haematological assessment and therapy [15].

## 5. Conclusions

Co-infections of DENV and SARS-CoV-2 had notably heightened incidences of WHO 1997 DHF/DSS, WHO 2009 SD and SD warning sign criteria. Importantly, individuals who were previously infected with SARS-CoV-2 before DENV infections exhibited significantly increased incidences of thrombocytopenia (as per DHF/DSS criteria) as well as several SD warning signs. These findings underscore the critical importance for healthcare professionals to further evaluate the incidence of DENV-SARS-CoV-2 co-infections and question DENV-infected patients about their previous incidence of SARS-CoV-2 infections and apply both the WHO 1997 and 2009 DHF/DSS/SD and SD warning sign criteria to design and provide optimal treatment.

## Figures and Tables

**Table 1:** General characteristics and clinical features of the study population who had confirmed DENV infections before the COVID-19 pandemic (2018–2019) compared to during the COVID-19 pandemic (2020–2022).

		SARS-CoV-2-Co-Infected (*n* = 517)		Non-SARS-CoV-2-Coinfected (*n* = 1089)		*p*-Value
		*n*	%	*n*	%	
Gender	Male	253	48.94	558	51.24	<0.001
	Female	264	51.06	531	48.76	<0.001
Age Group	18–40 years old	370	71.57	827	75.94	<0.001
	41–60 years old	102	19.73	179	16.44	0.000
	>60 years old	45	8.70	82	7.53	<0.001
Stratum	1	127	24.56	314	28.83	<0.001
	2	185	35.78	378	34.71	<0.001
	3	97	18.76	191	17.54	0.001
	4	57	11.03	117	10.74	<0.0001
	5	22	4.26	31	2.85	0.1655
	6	6	1.16	12	1.10	0.1573
Patient Hospitalized	Yes	260	50.29	599	55.00	<0.0001
	No	257	49.71	490	45.00	<0.0001
Symptoms	Fever	517	100.00	1089	100.0	<0.0001
	Headache	450	87.04	941	86.41	<0.0001
	Retro-orbital Pain	235	45.45	514	47.20	<0.0001
	Myalgia	427	82.59	910	83.56	<0.0001
	Arthralgia	389	75.24	802	73.65	<0.0001
	Rash	162	31.33	321	29.48	0.005
	Abdominal Pain	174	33.66	377	34.62	<0.0001
	Vomiting	90	17.41	232	21.30	<0.0001
	Diarrhoea	68	13.15	143	13.13	<0.0001
	Drowsiness	12	2.32	52	4.78	<0.0001
	Hypotension	28	5.41	37	3.39	0.0005
	Hepatomegaly	5	0.97	11	1.01	0.1336
	Bloody Mucus	7	1.35	26	2.39	0.0009
	Haemoconcentration	22	4.26	43	3.95	0.0091
	Dyspnoea	178	34.4	61	5.6	<0.0001
	Reduced Platelet Numbers	201	38.88	417	38.29	<0.0001
	Liquid Accumulation	10	1.93	20	1.84	0.0678

*n*: Total cases, %: percentages. Socio-economic stratum: 1: low, 2: low-medium, 3: medium, 4: upper medium, 5: high-medium, 6: high.

**Table 2:** Demographic and clinical features of confirmed DENV-SARS-CoV-2 co-infections and non-coinfected cases.

		SARS-CoV-2-Co-Infected (*n* = 517)		Non-SARS-CoV-2-Coinfected (*n* = 1089)		*p*-Value
		*n*	%	*n*	%	
Gender	Male	253	48.94	558	51.24	<0.001
	Female	264	51.06	531	48.76	<0.001
Age Group	18–40 years old	370	71.57	827	75.94	<0.001
	41–60 years old	102	19.73	179	16.44	0.000
	>60 years old	45	8.70	82	7.53	<0.001
Stratum	1	127	24.56	314	28.83	<0.001
	2	185	35.78	378	34.71	<0.001
	3	97	18.76	191	17.54	0.001
	4	57	11.03	117	10.74	<0.0001
	5	22	4.26	31	2.85	0.1655
	6	6	1.16	12	1.10	0.1573
Patient Hospitalized	Yes	260	50.29	599	55.00	<0.0001
	No	257	49.71	490	45.00	<0.0001
Symptoms	Fever	517	100.00	1089	100.0	<0.0001
	Headache	450	87.04	941	86.41	<0.0001
	Retro-orbital Pain	235	45.45	514	47.20	<0.0001
	Myalgia	427	82.59	910	83.56	<0.0001
	Arthralgia	389	75.24	802	73.65	<0.0001
	Rash	162	31.33	321	29.48	0.005
	Abdominal Pain	174	33.66	377	34.62	<0.0001
	Vomiting	90	17.41	232	21.30	<0.0001
	Diarrhoea	68	13.15	143	13.13	<0.0001
	Drowsiness	12	2.32	52	4.78	<0.0001
	Hypotension	28	5.41	37	3.39	0.0005
	Hepatomegaly	5	0.97	11	1.01	0.1336
	Bloody Mucus	7	1.35	26	2.39	0.0009
	Haemoconcentration	22	4.26	43	3.95	0.0091
	Dyspnoea	178	34.4	61	5.6	<0.0001
	Reduced Platelet Numbers	201	38.88	417	38.29	<0.0001
	Liquid Accumulation	10	1.93	20	1.84	0.0678

*n:* Total cases, %: percentages, *p*-value: probability value. Socio-economic stratum: 1: low, 2: low-medium, 3: medium, 4: upper medium, 5: high-medium, 6: high.

**Table 3:** Clinical characteristics of DENV-infected patients associated with SARS-CoV-2 co-infection using logistic regression analysis.

Variables	Bivariate Analysis		Multivariate Analysis		Multivariate Analysis (Age-Adjusted Model)	
	Crude OR	(95% CI)	Adj. OR	(95% CI)	Adj. OR	(95% CI)
Fever	1.55	(1.15, 2.08)	1.47	(1.07, 2.02)	1.67	(1.19, 2.06)
Headache	1.62	(1.07, 2.45)	1.58	(1.01, 2.48)	1.72	(1.0, 2.99)
Myalgia	1.65	(1.13, 2.4)	2.09	(1.73–2.53)	-	-
Abdominal Pain	1.85	(1.37, 2.5)	3.41	(2.73–4.28)	-	-
Arthralgia	1.7	(1.22, 2.35)	1.5	(1.04, 2.18)		
Fatigue	1.78	(1.48, 2.15)	-	-	2.01	(1.54, 2.61)
Dyspnoea	1.27	(1.06, 1.53)	1.31	(1.11, 1.68)	1.47	(1.14, 1.89)
Hypotension	1.34	(1.11, 1.61)	1.4	(1.15, 1.71)	1.52	(1.18, 1.96)
Haemoconcentration	1.22	(1.02, 1.46)	1.31	(1.08, 1.6)	1.42	(1.1, 1.82)
Laboratory findings						
Platelets 10^9^/L	1.79	(1.32, 2.43)	1.45	(1.08, 1.94)	1.76	(1.15, 2.71)
WBC	1.1	(0.91, 1.31)	-	-	-	-
ALT	1.72	(1.31, 2.24)	0.66	(0.48, 0.91)	0.98	(0.65, 1.47)
AST	1.23	(1.04, 1.44)	0.55	(0.44, 0.67)	0.91	(0.71, 1.17)

Crude OR: crude odds ratio; (95% CI): 95% confidence interval; Adj. OR: adjusted odds ratio.

**Table 4:** Clinical characteristics associated with DHF/DSS/SD or SD warning signs using logistic regression analysis.

	B	S.E.	*p*-Value	OR	95% CI for OR	
					Lower	Upper
Fever						
No	0.00 (Ref.)	0	1.00	1.00 (Ref.)	1	1
Yes	0.467	0.1815	0.010	1.6	1.12	2.29
Headache						
No	0.00 (Ref.)	0	1.00	1.00 (Ref.)	1	1
Yes	0.6763	0.1325	<0.001	1.97	1.52	2.55
Retro-orbital Pain						
No	0.00 (Ref.)	0	1.00	1.00 (Ref.)	1	1
Yes	−1.7601	0.1086	<0.001	0.17	0.14	0.21
Myalgia						
No	0.00 (Ref.)	0	1.00	1.00 (Ref.)	1	1
Yes	1.5204	0.1303	<0.001	4.57	3.55	5.93
Arthralgia						
No	0.00 (Ref.)	0	1.00	1.00 (Ref.)	1	1
Yes	0.3627	0.1081	0.0007	1.44	1.16	1.78
Platelets						
Normal	0.00 (Ref.)	0	1.00	1.00 (Ref.)	1	1
Reduced	0.549	0.1018	<0.001	1.73	1.42	2.12
Abdominal Pain						
No	0.00 (Ref.)	0	1.00	1.00 (Ref.)	1	1
Yes	−1.8237	0.1224	<0.001	0.16	0.13	0.2
Dyspnoea						
No	0.00 (Ref.)	0	1.00	1.00 (Ref.)	1	1
Yes	0.419	0.1069	0.005	1.8	1.1	2.35
Haemoconcentration						
No	0.00 (Ref.)	0	1.00	1.00 (Ref.)	1	1
Yes	0.1182	0.2607	0.0250	1.32	1.18	1.57
SARS-CoV-2 infection						
No	0.00 (Ref.)	0	1.00	1.00 (Ref.)	1	1
Yes	0.8064	0.1590	<0.001	1.45	1.30	1.59

B: coefficient value; S.E.: standard error; *p*-value: probability value; OR: odds ratio; 95% CI for OR: 95% confidence interval for odds ratio; Lower: lower confidence interval value; Upper: upper confidence interval value.

## Data Availability

All data will be made available upon request through the corresponding author.

## Supplementary Materials

The following supporting information can be downloaded at: https://www.mdpi.com/article/10.3390/pathogens13070573/s1, Table S1: Clinical Chracteristics of the Non-severe (DF) or Severe (DHF/DSS/SD) DENV-infected Patients; Table S2: DENV-infected Patients’ Laboratory Results from Non-severe (DF) and Severe (DHF/DSS/SD) cases; Table S3: Clinical characteristics of IgM-capture ELISA-confirmed DENV infected patients with or without Previously-Confirmed SARS-CoV-2 infections; Table S4: Collinearity analysis in the multiple logistic regression co-infection model; Table S5: Collinearity Analysis Results of the Multiple Logistic Regression Age-adjusted Co-infection Model; Table S6: Random Forest (RF), Decision Tree (DT) Performance during the Training and Testing Stages; Table S7. Clinical Symptoms of DENV infected Patients with Previously Confirmed SARS-CoV-2 infections; Table S8: Collinearity Analysis in the Multiple Logistic Regression of DENV Infected Patients with Previous SARS-CoV-2 Infections.
