# Supplementary material for: Pre-or co-SARS-CoV-2 Infections Significantly Increase Severe Dengue Virus Disease Criteria: Implications for Clinicians
Source: Pathogens. 2024 Jul 10;13(7):573. doi: 10.3390/pathogens13070573 (PMC11279652; doi:10.3390/pathogens13070573)
Supplement: Supplementary file 1 [file pathogens-13-00573-s001.zip › pathogens-2965176-supplementary.pdf]

Table S1: Clinical Chracteristics of the Non-severe (DF) or Severe (DHF/DSS/SD) DENV-infected Patients

	Non-severe (DF) (n=2902)	Severe (DHF/DSS/SD) (n=709)	P-value
**Fever**			1.00
**Yes**	2902 (100)	709 (100)	
**No**	0	0	
**Headache**			0.11
**Yes**	2499 (86.1)	615(86.7)	
**No**	403 (13.9)	94 (13.3)	
**Retro-orbital Pain**			1.00
**Yes**	1356 (46.7)	330 (46.5)	
**No**	1546 (53.3)	379 (53.5)	
**Myalgia**			0.19
**Yes**	2398 (82.6)	576 (81.2)	
**No**	504 (17.4)	133 (18.8)	
**Arthralgia**			0.12
**Yes**	2116 (72.9)	497 (70.1)	
**No**	786 (27.1)	212 (29.9)	
**Rash**			0.1
**Yes**	898 (30.9)	216 (30.3)	
**No**	2004 (69.1)	493 (69.7)	
**Abdominal Pain**			<0.01
**Yes**	967 (33.4)	355 (50.2)	
**No**	1934 (66.6)	354 (49.8)	
**Vomiting**			0.04
**Yes**	628 (21.6)	244 (34.4)	
**No**	2273 (78.3)	463 (65.3)	
**Diarrhoea**			0.05
**Yes**	341 (11.88)	139 (19.6)	
**No**	2561 (88.2)	570 (80.4)	
**Drowsiness**			0.08
**Yes**	82 (2.8)	48 (6.8)	
**No**	2820 (97.2)	661 (93.2)	
**Hypotension**			0.04
**Yes**	201 (6.9)	94 (13.3)	
**No**	2701 (93.1)	615 (86.7)	
**Hepatomegaly**			0.04
**Yes**	218 (7.5)	89 (12.6)	
**No**	2684 (92.5)	620 (87.4)	
**Dyspnoea**			<0.01
**Yes**	470 (16.2)	244 (34.4)	
**No**	2432 (83.8)	465 (65.6)	
**Bloody Mucus**			0.03
**Yes**	105 (3.6)	77 (10.9)	
**No**	2797 (96.4)	632 (89.1)	

**Haemoconcentration**			0.05
**Yes**	518 (17.8)	108 (15.2)	
**No**	2384 (82.2)	601 (84.8)	
**Fluid Accumulation**			0.19
**Yes**	164 (5.7)	42 (5.9)	
**No**	2738 (94.3)	667 (94.1)	
***total cases (percentages) ; P-value: probability value**			

Table S2: DENV-infected Patients' Laboratory Results from Non-severe (DF) and Severe (DHF/DSS/SD) cases

	**Overall**	**Non-severe**	**Severe**	**P-value**
	**(N=3611)**	**(N=2902)**	**(N=709)**	
**Platelet Numbers (10^9/L)**				0.01
**Reduced**	2208 (61.1)	1571 (54.1)	637 (89.8)	
**Within normal range**	1403 (38.9)	1331 (45.9)	72 (10.2)	
**Missing**	0	0	0	
**WBC Numbers (10^9/L)**				<0.001
**Reduced**	833 (23.1)	689 (23.7)	144 (20.3)	
**Within normal range**	2313 (64.1)	1845 (63.6)	468 (66.0)	
**Missing**	465 (12.9)	368 (12.7)	97 (13.7)	
**ALT concentrations (U/L)**				0.16
**Raised**	779 (21.0)	638 (22.0)	121 (17.1)	
**Within normal range**	475 (13.2)	382 (13.2)	93 (13.1)	
**Not done**	2061 (57.1)	1641 (56.5)	420 (59.2)	
**Missing**	296 (8.2)	241 (8.3)	55 (7.8)	
**AST concentrations (U/L)**				0.009
**Raised**	689 (19.1)	565 (19.5)	124 (17.5)	
**Within normal range**	451 (12.5)	373 (12.9)	78 (11.0)	
**Not done**	2015 (55.8)	1600 (55.1)	415 (58.5)	
**Missing**	456 (12.6)	364 (12.5)	92 (13.0)	

WBC: white blood cell;
ALT: alanine transaminase
AST: aspartate transaminase.
P-value : Probability value

Table S3: Clinical characteristics of IgM-capture ELISA-confirmed DENV infected patients with or without Previously-Confirmed SARS-CoV-2 infections.

Symptoms	SARS-CoV-2 Negative (n=203)		SARS-CoV-2 positive (n=566)		P-value
	n	%	n	%	
**Fever**	203	100.00	566	100.00	< 0.001
**Headache**	171	84.24	478	84.45	0.0005
**Retro-orbital Pain**	69	33.99	259	45.76	0.081
**Myalgias**	170	83.74	439	77.56	0.0001
**Arthralgia**	159	78.33	389	68.73	0.0005
**Rash**	52	25.62	191	33.75	0.355
**Abdominal Pain**	77	37.93	178	31.45	0.0009
**Vomiting**	48	23.65	113	19.96	0.0001
**Diarrhoea**	27	13.30	57	10.07	0.0268
**Drowsiness**	11	5.42	21	3.7	0.0325
**Hypotension**	12	5.91	71	12.5	0.0376
**Hepatomegaly**	2	0.99	4	0.71	0.563
**Bloody Mucus**	6	2.96	6	1.06	--
**Dyspnoea**	56	27.58	183	32.3	<0.001
**Haemoconcentration**	21	10.34	84	14.8	0.0419
**Increased Haematocrit**	6	2.96	14	2.47	0.5271
**Reduced Platelets**	120	59.11	199	35.16	0.005
**Fluid Accumulation**	4	1.97	32	5.65	0.0520
**p-value: probability value**					

Table S4: Collinearity analysis in the multiple logistic regression co-infection model

	Factors	VIF
**1**	**Fever**	1.09
**2**	**Headache**	1.48
**3**	**Myalgia**	1.12
**4**	**Abdominal Pain**	1.22
**5**	**Arthralgia**	1.12
**6**	**Fatigue**	1.01
**7**	**Dyspnoea**	1.71
**8**	**Hypotension**	1.16
**9**	**Haemoconcentration**	1.24
**10**	**Platelet Numbers (10^9/µL)**	1.25
**11**	**WBC Numbers**	1.08
**12**	**ALT concentration**	1.09
**13**	**AST concentration**	1.27

Table S5: Collinearity Analysis Results of the Multiple Logistic Regression Age-adjusted Co-infection Model

	**Factors**	**VIF**
**1**	Fever	1.32
**2**	Headache	1.26
**3**	Fatigue	1.50
**4**	Dyspnoea	1.21
**5**	Hypotension	1.03
**6**	Haemoconcentration	1.26
**7**	Platelets (10^9/μL)	3.88
**8**	WBC	1.00
**9**	ALT	1.01
**10**	AST	1.13

Table S6: Random Forest (RF), Decision Tree (DT) Performance during the Training and Testing Stages.

**Stage**	**Training results**		**Testing results**	
**Model**	**RF**	**DT**	**RF**	**DT**
**Positive Predictive Value**	0.85	0.86	0.84	0.79
**Negative Predictive Value**	0.90	0.87	0.86	0.81
**Sensitivity**	0.92	0.89	0.91	0.78
**Specificity**	0.82	0.83	0.78	0.82
**Accuracy**	0.88	0.86	0.85	0.80
**AUC**	0.92	0.90	0.90	0.86

AUC: Area under curve

Table S7. Clinical Symptoms of DENV infected Patients with Previously Confirmed SARS-CoV-2 infections.

	B	S.E.	P value	OR	95% CI for OR	
					Lower	Upper
**Fever**						
**No**	0.00 (Ref.)	0	1	1.00 (Ref.)	1	1
**Yes**	0.9498	0.2321	<0.001	2.59	1.64	4.07
**Headache**						
**No**	0.00 (Ref.)	0	1	1.00 (Ref.)	1	1
**Yes**	1.4935	0.2909	0.001	4.25	2.52	7.88
**Myalgia**						
**No**	0.00 (Ref.)	0	1	1.00 (Ref.)	1	1
**Yes**	0.6046	0.2639	0.015	1.83	1.09	3.07
**Arthralgia**						
**No**	0.00 (Ref.)	0	1	1.00 (Ref.)	1	1
**Yes**	0.6629	0.2471	0.009	1.94	1.2	3.15
**Abdominal Pain**						
**No**	0.00 (Ref.)	0	1	1.00 (Ref.)	1	1
**Yes**	0.6765	0.3172	0.036	1.97	1.06	3.66
**Dyspnoea**						
**No**	0.00 (Ref.)	0	1	1.00 (Ref.)	1	1
**Yes**	0.5598	0.141	<0.001	1.75	1.33	2.31
**Rash**						
**No**	0.00 (Ref.)	0	1	1.00 (Ref.)	1	1
**Yes**	0.6464	0.2558	0.001	1.91	1.16	3.15
**Reduced Platelets**						
**No**	0.00 (Ref.)	0	1	1.00 (Ref.)	1	1
**Yes**	0.5809	0.282	<0.001	1.79	1.03	3.11
**Haemoconcentration**						
**No**	0.00 (Ref.)	0	1	1.00 (Ref.)	1	1
**Yes**	0.5273	0.1422	< 0.001	1.69	1.28	2.24
**Hypotension**						
**No**	0.00 (Ref.)	0	1	1.00 (Ref.)	1	1
**Yes**	0.3543	0.1414	0.011	1.43	1.08	1.88

coefficient value; S.E.: standard error; P-value: Probability value; OR: Odds Ratios; 95% CI for OR: 95% Confidence interval for Odds Ratios; Lower: Lower confidence interval value; Upper: Upper confidence interval value.

Table S8: Collinearity Analysis in the Multiple Logistic Regression of DENV Infected Patients with Previous SARS-CoV-2 Infections.

	Factors	VIF
**1**	Fever	1.03
**2**	Headache	1.17
**3**	Myalgia	1.12
**4**	Arthralgia	1.15
**5**	Abdominal Pain	1.15
**6**	Dyspnoea	1.03
**7**	Rash	1.04
**8**	Reduce Platelets	1.17
**9**	Haemoconcentration	1.05
**10**	Hypotension	1.03
